# Pharmacological fingerprint of antipsychotic drugs at the serotonin 5-HT_2A_ receptor

**DOI:** 10.1038/s41380-024-02531-7

**Published:** 2024-04-02

**Authors:** Supriya A. Gaitonde, Charlotte Avet, Mario de la Fuente Revenga, Elodie Blondel-Tepaz, Aida Shahraki, Adrian Morales Pastor, Valerij Talagayev, Patricia Robledo, Peter Kolb, Jana Selent, Javier González-Maeso, Michel Bouvier

**Affiliations:** 1 Institute for Research in Immunology and Cancer (IRIC), Department of Biochemistry and Molecular Medicine, Université de Montréal, Montréal, QC H3T 1J4 Canada; 2 Department of Physiology and Biophysics, School of Medicine, Virginia Commonwealth University, Richmond, VA 23298 USA; 3 Department of Pharmaceutical Chemistry, Philipps-Universität Marburg, Marbacher Weg 8, 35032 Marburg, Germany; 4 Research Programme on Biomedical Informatics (GRIB), IMIM-Hospital del Mar Medical Research Institute, Barcelona, 08003 Spain; 5 Integrative Pharmacology and Systems Neuroscience Research Group, IMIM-Hospital del Mar Medical Research Institute, Barcelona, 08003 Spain

**Keywords:** Drug discovery, Biological techniques, Schizophrenia

## Abstract

The intricate involvement of the serotonin 5-HT_2A_ receptor (5-HT_2A_R) both in schizophrenia and in the activity of antipsychotic drugs is widely acknowledged. The currently marketed antipsychotic drugs, although effective in managing the symptoms of schizophrenia to a certain extent, are not without their repertoire of serious side effects. There is a need for better therapeutics to treat schizophrenia for which understanding the mechanism of action of the current antipsychotic drugs is imperative. With bioluminescence resonance energy transfer (BRET) assays, we trace the signaling signature of six antipsychotic drugs belonging to three generations at the 5-HT_2A_R for the entire spectrum of signaling pathways activated by serotonin (5-HT). The antipsychotic drugs display previously unidentified pathway preference at the level of the individual Gα subunits and β-arrestins. In particular, risperidone, clozapine, olanzapine and haloperidol showed G protein-selective inverse agonist activity. In addition, G protein-selective partial agonism was found for aripiprazole and cariprazine. Pathway-specific apparent dissociation constants determined from functional analyses revealed distinct coupling-modulating capacities of the tested antipsychotics at the different 5-HT-activated pathways. Computational analyses of the pharmacological and structural fingerprints support a mechanistically based clustering that recapitulate the clinical classification (typical/first generation, atypical/second generation, third generation) of the antipsychotic drugs. The study provides a new framework to functionally classify antipsychotics that should represent a useful tool for the identification of better and safer neuropsychiatric drugs and allows formulating hypotheses on the links between specific signaling cascades and in the clinical outcomes of the existing drugs.

## Introduction

Schizophrenia is a chronic, debilitating condition with an unknown etiology [1] affecting about 1% of the population worldwide. Antipsychotic drugs remain the mainstay of treatment, and are classified into typical (ex: haloperidol), atypical (ex: clozapine, olanzapine and risperidone) and third generation (ex: aripiprazole and cariprazine) based on their broad mechanism of action and side effect profile [2]. The higher affinity of atypical antipsychotics at the serotonin 5-HT_2A_ receptor (5-HT_2A_R) compared to the dopamine D_2_ receptor has been historically considered relevant for their lower tendency of causing extra pyramidal side effects (EPS) [3–5]. In addition, whereas activity at the dopamine D_2_ receptor seems to be a prerequisite for effective antipsychotic activity, there is extensive evidence pointing towards the involvement of the 5-HT_2A_R, not only in the mechanism of action of antipsychotics, but also in the pathophysiology of schizophrenia [6–9]. For instance, activation of the 5-HT_2A_R by hallucinogenic compounds such as LSD is known to cause psychotic states similar to the positive symptoms of schizophrenia [10]. Moreover, studies over the years have detected changes in the expression levels of the 5-HT_2A_R in patients with schizophrenia [9]; in fact the specific upregulation of the signaling via the 5-HT_2A_R component of the 5-HT_2A_R-mGlu_2_R heterocomplex has been demonstrated to be an important contributing factor in schizophrenia symptoms [11–14]. Taking a step further, recent studies with postmortem samples of schizophrenia patients have identified functional selectivity at the G protein level for the 5-HT_2A_R, with heightened signaling via the inhibitory Gα_i1_ proteins versus the Gα_q_ pathway [15].

The past two decades have seen an increasing appreciation of the therapeutic ramifications of functional selectivity across GPCRs with drug discovery efforts being directed towards teasing out the pathways leading to beneficial effects versus those causing side effects [16–24]. While the focus has been primarily between the G protein and β-arrestin pathways, bias among the different G protein subtypes is being uncovered as well [20, 25–27]. While current antipsychotic drugs such as clozapine, risperidone and olanzapine are known inverse agonists at the 5-HT_2A_R [28, 29], little is known about their detailed functional selectivity profile at this critical receptor for schizophrenia treatment. A deeper understanding at the level of the different signaling pathways activated by serotonin (5-HT) could give an insight into their mechanisms of action and help shed some light on the pathophysiology of the disorder. Herein, with the suite of “Effector membrane translocation assay” (EMTA) enhanced bystander BRET (ebBRET) biosensors [20], we present the pharmacological fingerprint of six currently marketed antipsychotic drugs at the 5-HT_2A_R with the aim of unveiling critical information on their mode of action and to set the stage for the development of safer, more efficacious antipsychotic drugs. The study also revealed characteristic signaling fingerprints corresponding to the three classes of antipsychotics and provides some insights into the pathways that may be underlying specific side effects.

## Materials and methods

### Reagents

Serotonin (5-HT), olanzapine and aripiprazole were purchased from Sigma-Aldrich, risperidone, clozapine and cariprazine were purchased from Cayman Chemical Company and haloperidol was purchased from Tocris bioscience. The BRET^2^ substrates, coelenterazine 400a or DeepBlueC and [methoxy e-Coelenterazine (Me-O-e-CTZ)] or Prolume Purple were from Nanolight^TM^ Technology.

### Plasmids and cell culture

The human 5-HT_2A_R was a gift from Domain Therapeutics North America. The human untagged G protein subunits were purchased from cdna.org. The following BRET-based biosensor components have been previously described: human G protein subunits, GRK2-D110A-GFP10, GRK2-GFP10, Gγ5-RlucII [30]; p63-RlucII [20], Rap1GAP-RlucII [20]; PDZ-RlucII [20]; Gα_s_67-RlucII [31], β-arrestin1-RlucII, β-arrestin2-RlucII, rGFP-CAAX [25, 32].

All the experiments presented in this study were performed in HEK293 SL (hereafter named HEK293) clonal cell line, a gift from S. Laporte (McGill University, Montreal, Quebec, Canada), and have been described in [32], except in experiments comparing HEK293 cells backgrounds, where HEK293 T cells and the derived Bcm3 clonal cell line developed in Dr Bouvier’s laboratory [33] were also used. Dulbecco’s Modified Eagle Medium (DMEM), trypsin, newborn calf serum (NCS), fetal bovine serum (FBS), antibiotics [penicillin and streptomycin (PS)] and all other cell culture reagents were purchased from Wisent Inc. All cell lines were regularly tested for mycoplasma contamination.

### Transfection

HEK293 cells were maintained in DMEM supplemented with 10% (v/v) NCS, 1% (v/v) antibiotics (100 U/mL penicillin and 100 μg/mL streptomycin; PS) and cultured in T150 cm^2^ flasks (Corning®) at 37 °C with 5% CO_2_ and 90% humidity. DNA mixtures (1000 ng adjusted with salmon sperm DNA; Invitrogen) were prepared in phosphate buffered saline (PBS; pH 7.4). Polyethyleneimine (PEI; Polysciences, Inc.) used for transfection was diluted in PBS, at a PEI:DNA ratio of 3:1, and was added to the DNA at least 15 min before addition of the DNA mix to the cells. A cell suspension of 3.5 × 10^5^ cells/mL in DMEM was prepared and added to the DNA-PEI transfection mix, and immediately distributed (3.5 × 10^4^ cells/100 µl/well) in 96-well white microplates (Greiner Bio-One) precoated with poly-L-ornithine (Sigma-Aldrich). The cells were maintained in culture for 48 h before conducting BRET experiments, except for the test evaluating whether 5-HT in the serum interfere with the activity of the receptor for which the cells were starved 24 h before BRET assay in 2% NCS (instead of 10% NCS normally). To determine receptor expression levels, saturation radioligand binding experiments were performed using [^3^H]MDL100907 as previously reported [34]. The amount of 5-HT_2A_R transfected across the different biosensors tested was the same and the receptor level determined to be 1675 fmol/mg protein.

To determine the G protein-activation profile of 5-HT across an entire panel of wild-type Gα subunits with the GRK2 biosensor, cells were transfected with the receptor, the respective Gα subunits, Gβ_1_, Gγ_5_-RlucII and GRK2-D110-GFP10 or GRK2-GFP10. To detect activation of a G protein pathway, cells were transfected with the receptor, the Gα subunit, and rGFP-CAAX along with either p63-RlucII, Rap1GAP-RlucII, PDZ-RlucII or Gα_s_67-RlucII, depending on the Gα family being tested. For activation of Gα_s_, Gβ_1_ and Gγ_1_ were co-transfected along with Gα_s_67-RlucII. For recruitment of the β-arrestins, cells were transfected with the receptor, β-arrestin1-RlucII or β-arrestin2-RlucII, GRK2 and rGFP-CAAX.

### Bioluminescence resonance energy transfer (BRET) assays

On the day of the BRET experiments, the cell culture medium was removed, and the cells were washed twice with PBS and treated with Tyrode’s buffer (140 mM NaCl, 2.7 mM KCl, 1 mM CaCl_2_, 12 mM NaHCO_3_, 5.6 mM d-glucose, 0.5 mM MgCl_2_, 0.37 mM NaH_2_PO_4_, 25 mM HEPES [pH 7.4]) and incubated at 37 °C for at least 15 min. To validate absence of effect from potential 5-HT present in the standard serum, cells were starved the night before the BRET assay in DMEM medium containing 2% of NCS (instead of 10% for regular experiments).

For the GRK2 recruitment-based biosensor used to establish which G proteins are engaged by the 5-HT_2A_R, cells were treated with a supramaximal concentration of 5-HT and the ligands, incubated for 10 min after which the substrate coelenterazine 400a (2.5 µM) was added and the cells were incubated for 5 min before reading the BRET signal on the Spark® multimode microplate reader (Tecan) (acceptor, 515 ± 20 nm; and donor, 400 ± 70 nm filters).

Antipsychotics were tested in their agonist mode (in the absence of 5-HT) and antagonist mode (in the presence of 5-HT at its EC_80_) using the EMTA biosensor suite with the same wavelength as above. For the concentration response curves of 5-HT and the ligands, cells were stimulated with different concentrations of the ligands and incubated for 5 min after which the substrate Prolume Purple (1.3 µM) was added and incubated for an additional 6 min. BRET signal was measured on the Spark® multimode microplate reader (Tecan) (agonist mode). Cells were thereafter treated with a submaximal concentration of 5-HT (EC_80_ concentration at the respective pathways) and incubated for an additional 5 min after which a second BRET read (antagonist mode) was conducted. To assess the role of Gα_i/o_ proteins in 5-HT-mediated responses, cells were pretreated with pertussis toxin (PTX;100 ng/mL, 18 h; List Biological Laboratories) before agonist stimulation.

### Computational analyses

To do the principal component analysis (PCA), signal transduction data was arranged in a 2-dimensional array where each pathway and metric pair was placed in the columns and drugs were arranged in rows. Each metric, i.e. IC_50_ and % of inhibition were scaled between 0 and 1 to eliminate the impact of the difference in range between them. After that, each drug was projected into the first 2 principal components of the row vectors using the python module scikit-learn.decomposition.

### Docking studies

Various known structures of the serotonin 5-HT_2A_R, including 6A93, 6A94, 7VOE, 7VOD, 6WHA and 7RAN were prepared using the protein preparation menu of Molecular Operating Environment (MOE) software, which is an integrated software package for drug discovery [35]. 3D structures of the six ligands were downloaded as mol2 files from the ZINC15 database at pH 7.4 [36]. In order to increase the variety of available poses, docking runs were conducted with either DOCK [37] or with HYBRID [38], which is included in the OEDocking suite (OEDOCKING 4.1.2.1: OpenEye Scientific Software, Inc., Santa Fe, NM. http://www.eyesopen.com). For docking calculations in HYBRID, receptors were prepared with pdb2receptor. Inputs for DOCK, which include receptor version with polar hydrogens, without hydrogens and a co-crystallized ligand in pdb format were prepared in MOE. Poses were visually selected for plausibility of binding mode (i.e. satisfaction of polar contacts, lack of intramolecular strain, absence of clashes with the receptor, etc). For consistency, the obtained poses were then subjected to energy minimization using the MMFF94x force field, which is a variant of MMFF94 (Merck Molecular force field) and is suitable for minimizing protein-ligand complexes [39] in MOE. During analysis, special attention was paid to the interaction with D3.32, as this residue is highly conserved among aminergic receptors. Risperidone in 6A93, aripiprazole in 7VOE and cariprazine in 7VOE were also minimized using the same force field. PyMOL (The PyMOL Molecular Graphics System, Version 2.0, Schrödinger, LLC) was used for visualization of the poses.

### Animals and drugs

C57BL/6 wild-type males, sourced from JAX farms, 11 weeks of age at the time of experiment, were housed in groups of up to five littermates with food and water *ad libitum* in a vivarium with a 12 h light/dark cycle at 23 °C. Animals were allowed to get accustomed to the vivarium at least 1 week prior to the experiment. Experiments were conducted in accordance with NIH guidelines and were approved by the Virginia Commonwealth University Animal Care and Use Committee. All efforts were made to minimize animal suffering and the number of animals used.

Risperidone and aripiprazole were sourced from Tocris bioscience. Risperidone was dissolved in saline 0.9% solution and aripiprazole in 10% DMSO-0.9% saline solution and administered intra-peritoneally (10 μL/g animal weight). The hydrochloride salts of risperidone and aripiprazole were formed in situ by addition of 1 eq. HCl. Aripiprazole was administered as a fine suspension.

### IP1 experiment

Animals (5 animals per group) were sacrificed by cervical dislocation 1 h after drug (risperidone 3 mg/Kg and aripiprazole 2 mg/Kg) or vehicle and processed as previously described [40]. Briefly, frontal cortices were harvested and homogenized in a 10 µL per 1 mg of tissue solution consisting of 10% IP-One Gq Kit Lysis and Detection Buffer in Stimulation Buffer. The clarified homogenates (17,000 × *g* for 15 min) were plated in duplicate by sequential addition of 2 µL to a 18 µL mix of detection reagents in Lysis and Detection Buffer. The plates (HTRF 96-well low volume white plate, Cisbio-PerkinElmer) were read (emission at 615 and 665 nm following excitation at 320 nm and a 70 µs delay) within min of mixing at rt in a VICTOR Nivo (PerkinElmer) plate reader. The ratio between emission at 615 nm/665 nm for all conditions was calculated relative to the vehicle condition to determine the fold-change in IP1 concentration in the original sample. Standards ranging from 0 to 1.1 µM IP1 were employed to ensure linearity in the working concentrations used.

### Data and statistical analyses

For in vitro experiments, curves fitting was conducted using GraphPad Prism 9 (version 9.0.0). Concentration response curves were obtained using a three-parameter logistic nonlinear regression model and the results are expressed as mean ± SEM of at least three independent experiments. Raw BRET data is defined as the ratio of the light intensity emitted by the acceptor (515 nm) over the light intensity emitted by the donor (410 nm) (rGFP/RlucII). Ligand-promoted BRET (ΔBRET) was determined by subtracting the BRET ratio of the vehicle condition from the BRET ratio of the ligand-treated conditions. For the agonist and antagonist modes, the ligand-promoted BRET was normalized with respect to the maximal response of 5-HT (% response of 5-HT), and BRET promoted by 5-HT at its EC_80_ concentration (5-HT(EC_80_)), respectively, to get the logEC_50_ and *E*_max_ and the logIC_50_ values.

The Cheng-Prusoff equation as modified by the Cheng 2002 paper [41] was used to calculate the equilibrium dissociation constant:

$${K}_{{{{{{\rm{B}}}}}}}=\frac{{{{{{{\rm{IC}}}}}}}_{50}}{{1+\left[\frac{{{{{{\rm{A}}}}}}}{{{{{{{\rm{EC}}}}}}}_{50}}\right]}^{K}}$$

where, IC_50_: is the concentration of the compounds producing 50% of the inhibition of the activation mediated by the EC_80_ concentration (A) of 5-HT. *K* is the slope of the inhibition curve of the compounds tested in the antagonist mode. EC_50_ is the concentration of 5-HT producing 50% of the maximal response.

For in vivo experiments, no specific statistical method was used to calculate sample size, which was based on previous experiences with the same assays and are in line with the state of the art for similar assays. The mice used in the studies came from the same colony and were split randomly between the experimental groups. The investigators were blinded to group allocation during compounds administration to mice. Data are presented as mean ± SEM of 5 mice for each treated group.

Statistical significance was assessed with GraphPad Prism 9 (version 9.0.0) by Student’s t-test (two-tailed) and ANOVA followed by Dunnett’s post hoc test for multiple comparisons, performed as appropriate (see figure legends). The level of significance was chosen at *p* < 0.05.

## Results

### Signaling profile of 5-HT at the 5-HT_2A_R

**Fig. 1:**
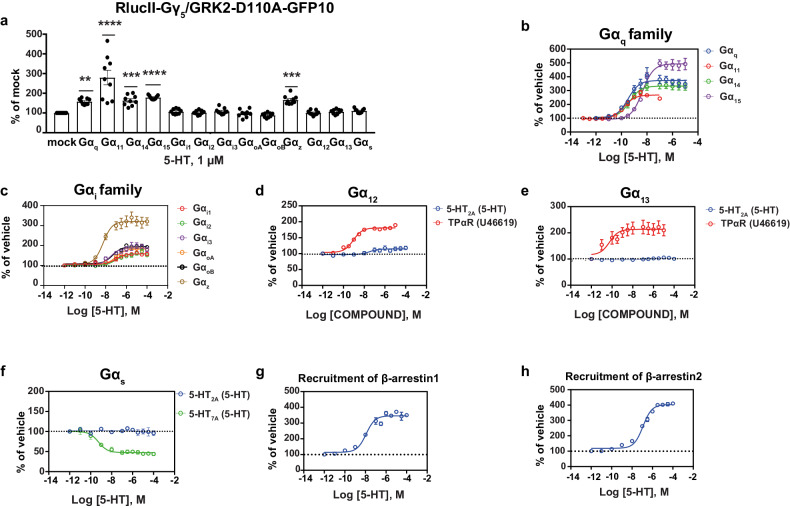
The complete signaling profile of 5-HT at the 5-HT_2A_R in HEK293 cells. **a** The complete G protein-activation profile of 5-HT (1 µM; 15 min) in HEK293 cells heterologously expressing the untagged 5-HT_2A_R and the biosensor (RlucII-G_γ5_/GRK2-D110A-GFP10, Gβ_1_ and the respective Gα subunits). Results are expressed as BRET ratio (GFP10/RlucII) as % of mock condition (in the absence of heterologously expressed Gα subunit) (mean ± SEM; *n* = 3; one-way ANOVA followed by Dunnett’s post hoc: **p = 0.0029, ***p = 0.0009, and ****p < 0.0001 compared to the mock condition). Concentration response curves showing the activation of the Gα_q_ family (**b**), Gα_i/o/z_ family (**c**), Gα_12_ (**d**), Gα_13_ (**e**), Gα_s_ (**f**), as well as the recruitment of β-arrestin1 (**g**) or β-arrestin2 (**h**) using the ebBRET-based EMTA biosensor in HEK293 cells heterologously expressing the untagged 5-HT_2A_R, the following biosensors (rGFP-CAAX along with p63-RlucII for Gα_q_, Rap1GAP-RlucII for Gα_i/o/z_, PDZ-RlucII for Gα_12_ and Gα_13_, Gα_s_67-RlucII for Gα_s_, β-arrestin1-RlucII or β-arrestin2-RlucII for β-arrestins), the respective Gα subunits (Gα_q_ family, Gα_i/o/z_ family, Gα_12/13_), Gβ_1_ and Gγ_1_ (Gα_s_) or WT-GRK2 (β-arrestins). The HA-TPα receptor (U46619) was used as the positive control for Gα_12/13_ while the 5-HT_7A_ receptor (5-HT) was used as the positive control for Gα_s_. Results are expressed as BRET ratio (rGFP/RlucII), as % over vehicle (mean ± SEM; *n* = 3).

As a first step in this study, we examined the complete G protein-activation profile of 5-HT at the predominantly Gα_q_-coupled 5-HT_2A_R in human embryonic kidney (HEK) 293 cells using a BRET^2^ biosensor to rapidly determine the G protein subtypes engaged by a receptor-ligand pair. This biosensor, as previously described [30, 42], allows screening for the activation of an entire panel of wild-type Gα subunits belonging to the different families; Gα_q_ (G_q_, G_11_, G_14_, G_15_), Gα_i/o/z_ (G_i1_, G_i2_, G_i3_, G_oA_, G_oB_, G_z_), Gα_12/13_ (G_12_, G_13_) and Gα_s_. The assay is based on the detection of BRET between a GRK2 construct fused to the blue-shifted green fluorescent protein GFP10 (GRK2-GFP10) and Gγ_5_ fused to luciferase from *Renilla reniformis* (RlucII-Gγ_5_). The increase in BRET upon G protein-activation reflects the dissociation of Gα from Gγ thus allowing an association between the released RlucII-Gγ_5_ and GRK2-GFP10. We used a mutant form of GRK2 (D110A), which lacks the RGS domain for Gα_q_, thus eliminating the possibility of a potential bias towards the detection of Gα_q_ activation. Upon stimulation with 5-HT we observed robust activation of all members of the Gα_q_ family (Gα_q_, Gα_11_, Gα_14_, Gα_15_) along with Gα_z_ from the Gα_i/o/z_ family (Fig. 1a). The results are comparable to those obtained using the wild-type (WT) form of GRK2-GFP10 (Supplementary Fig. 1a). Although this pattern of activation by 5-HT is in agreement with a recent study demonstrating the activation of mainly the Gα_q_ family and Gα_z_ with little or no activation of other Gα_i/o_ family members [43], the 5-HT_2A_R has also been reported to activate pathways downstream of the PTX-sensitive Gα_i/o_ family, especially with respect to the pathophysiology of schizophrenia and the mechanism of action of hallucinogenic drugs [15, 20, 44, 45]. In particular, inverse agonist activity at Gα_i1_ of the 5-HT_2A_R-selective ligand (pimavanserin) was recently reported in human postmortem brain samples and mice brain cortices [26].

Hence, we further investigated the activation of the Gα_i/o/z_ family using the more sensitive EMTA biosensors that measure the increase in BRET signal upon the recruitment of Gα family-specific effectors to the plasma membrane thus being a more direct reporter of the Gα activation state [20]. The assay used the Gα subunit selective effectors p63-RhoGEF for the Gα_q_ family, PDZ-RhoGEF for Gα_12/13_ and Rap1GAP for the Gα_i/o/z_ family as well as Gα_s_ itself fused to RlucII as the energy donor. The BRET between these donors and the *Renilla reniformis* GFP (rGFP) anchored at the inner face of the plasma membrane by the CAAX motif from Kras [20] is then used as an indicator of the G protein subtype activation. Similar to what was detected with the GRK2-based biosensor, we observed robust activation of the Gα_q_ family members and Gα_z_ (Fig. 1b, c). Moreover, we detected activation of the Gα_i/o_ subtypes (Fig. 1c), and this response was found to be sensitive to pertussis toxin (PTX), suggesting direct activation of the different Gα_i/o_ subunits by the 5-HT_2A_R (Supplementary Fig. 1b). It is noteworthy that the activity detected in the absence of heterologous expression of Gα subunit was significantly reduced by PTX, reflecting the activation of endogenously expressed Gα_i/o_. The remaining response observed in the presence of PTX most likely reflects activation of the endogenously expressed PTX- insensitive Gα_z_, consistent with the robust response observed using either the GRK2- or Rap1GAP-based biosensors upon heterologous expression of Gα_z_. Given that only weak coupling of 5-HT_2A_R to Gα_z_ *vs* other Gα_i/o_ family members had been previously reported [43], we compared the residual Gα_z_-mediated response following PTX treatment in three different HEK293 cell clones. As can be seen in Supplementary Fig. 1b, the PTX-resistant response represented between 40 ± 8 and 61 ± 7% of the global Rap1GAP response, indicating a cell type-dependent variation in the extent of Gα_z_ coupling that may result from different relative expression of the Gα_i/o/z_ subtypes in different clones. It should also be noted that Kim et al. used a BRET biosensor based on the dissociation between Gα and Gβγ subunits that, in contrast to the EMTA sensors used herein, requires modification of the Gα with the insertion of the energy donor Rluc in the Gα structure which may affect coupling and makes comparison between different Gα coupling difficult.

The activation of the Gα_q_ and Gα_i/o/z_ family observed in our study was entirely 5-HT_2A_R-dependent since no responses were detected in the absence of 5-HT_2A_R heterologous expression (Supplementary Fig. 1c). To exclude the possibility that 5-HT originating from the serum may interfere with the responses, experiments monitoring Gα_q_ activation were carried out in cells grown in media containing either 2% or 10% new calf serum (NCS). As shown in Supplementary Fig. 1d, no differences were observed, indicating that any possibly residual 5-HT did not interfere with the assay.

With the EMTA biosensor for Gα_12_ and Gα_13_ we confirm that the 5-HT_2A_R does not directly activate Gα_12_ or Gα_13_ (Fig. 1d, e), in contrast to the robust activation observed for the TPα receptor used as a positive control. Although earlier studies have implicated Gα_12/13_ in the activation of the phospholipase A_2_ (PLA_2_) pathway downstream of the 5-HT_2A_R, other studies have also shown that there is no evidence for a direct coupling of these G proteins by the 5-HT_2A_R [20, 43, 46]. No activation of Gα_s_ (indicated by a decrease in BRET signal as a result of Gα_s_67-RlucII leaving the plasma membrane decorated with the BRET acceptor rGFP as observed for 5-HT_7A_R used as a positive control) was observed for the 5-HT_2A_R upon 5-HT stimulation (Fig. 1f). In contrast to previously reported 5-HT-promoted cAMP accumulation in untransfected HEK293 cells [47], presumably due to the presence of endogenously expressed 5-HT_7A_R, we did not detect any 5-HT-promoted activation of Gα_s_ in untransfected cells (Supplementary Fig. 1e). This difference between the two studies may arise from different HEK293 clones being used or different sensitivities of the biosensors. In any case, no coupling of 5-HT_2A_R to Gα_s_ could be detected and this pathway was not further explored.

Activation of the 5-HT_2A_R by 5-HT leads to the recruitment of the β-arrestins as seen with the robust signal observed with the EMTA biosensors (β-arrestin1 and β-arrestin2 respectively fused to RlucII recruited to the plasma membrane decorated with rGFP) (Fig. 1g, h). The potency of 5-HT (logEC_50_ and logEC_80_) for the activation of the different G protein subtypes and β-arrestin recruitment is presented in Supplementary Table 1.

### Functional selectivity profile of antipsychotic drugs at the signaling pathways activated by 5-HT

**Fig. 2:**
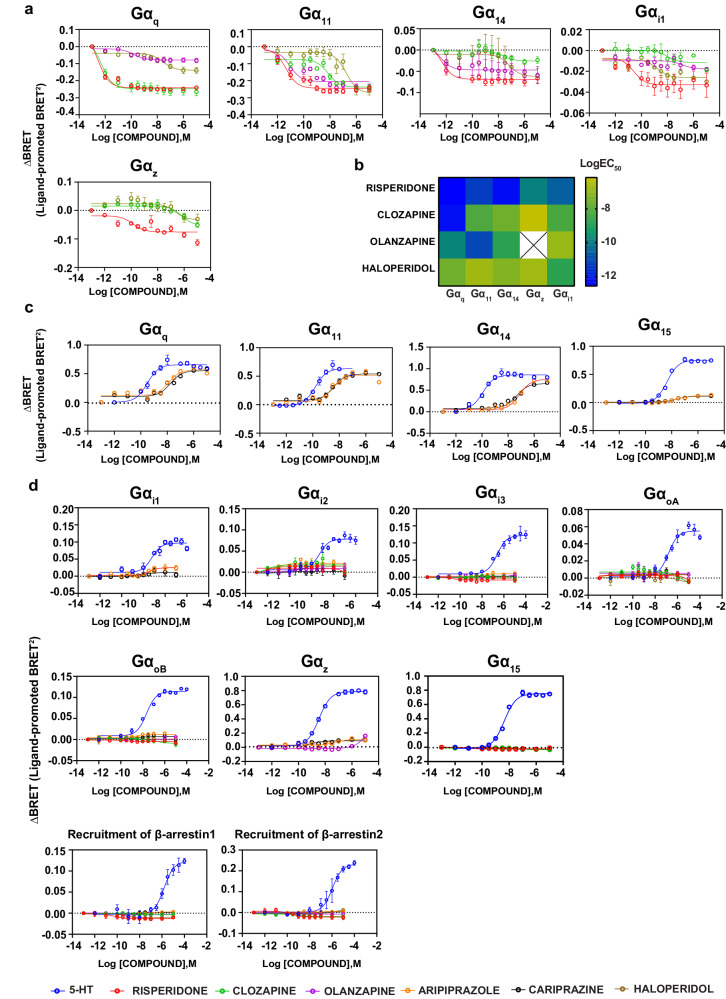
Differential agonist and inverse agonist activities of the antipsychotic drugs at the G protein pathways and for recruitment of β-arrestins. **a** Concentration response curves depicting the inverse agonist activities of risperidone, clozapine, olanzapine and haloperidol using the ebBRET-based EMTA biosensors in HEK293 cells heterologously expressing the untagged 5-HT_2A_R, the biosensor (rGFP-CAAX along with p63-RlucII for the Gα_q_ family and Rap1GAP-RlucII for Gα_i1/z_) and the respective Gα subunits. **b** Heatmap illustrating the potency (logEC_50_) of inverse agonist activity shown by risperidone, clozapine, olanzapine and haloperidol from the concentration response curves. The empty cell with a cross indicates no inverse agonist activity. **c** Concentration response curves depicting the partial agonist activities of aripiprazole and cariprazine at the Gα_q_ family using the ebBRET-based EMTA biosensors in HEK293 cells heterologously expressing the untagged 5-HT_2A_R, the biosensor (rGFP-CAAX along with p63-RlucII for the Gα_q_ family). **d** Curves of the six antipsychotic drugs at pathways where there was no activation when tested in the agonist mode using the ebBRET-based EMTA biosensors in HEK293 cells overexpressing the untagged 5-HT_2A_R, the biosensors (rGFP-CAAX along with p63-RlucII for the Gα_q_ family, Rap1GAP-RlucII for Gα_i/o/z_ family, β-arrestin-RlucII/WT-GRK2 for β-arrestins) and the respective Gα subunits (for Gα_q_ and Gα_i/o/z_ families). Results are expressed as ΔBRET (ligand-promoted BRET) (mean ± SEM; *n* = 3).

Based on the signaling profile of 5-HT, we tested six antipsychotic drugs (risperidone, clozapine, olanzapine, aripiprazole, cariprazine and haloperidol) for their activity at the Gα_q_ and Gα_i/o/z_ family members and for the recruitment of the β-arrestins. When tested in the agonist mode, each antipsychotic has a specific activation/inactivation profile, providing a unique signaling signature, i.e., pathway-preference profile, of their activity at the 5-HT_2A_R (Fig. 2). Our analyses reveal that the compounds can be divided into two groups, four having inverse agonist activity (risperidone, clozapine, olanzapine and haloperidol) and two being partial agonists (aripiprazole and cariprazine) on different pathways.

### Risperidone, clozapine, olanzapine and haloperidol demonstrate pathway preference in their inverse agonist activity

Although antipsychotics have been reported to have inverse agonist activity at the 5-HT_2A_R [29, 48], to our knowledge, this is the first study assessing this activity for a set of antipsychotics at all the G protein subtypes engaged by this receptor. Overall, risperidone, clozapine, olanzapine and haloperidol were found to be inverse agonists (i.e.: inhibiting the constitutive activity of the 5-HT_2A_R) mainly at Gα_q_, Gα_11_, Gα_14_, Gα_z_, and Gα_i1_. No inverse agonist activity was detectable toward the other Gα_i/o_ family members (G_i2_, G_i3_, G_oA_, G_oB_) or β-arrestin.

Among the four antipsychotics with inverse agonist activity, risperidone has the highest potency across different pathways (sub-picomolar and picomolar range at Gα_q_, Gα_11_ and Gα_14_; Fig. 2b and Supplementary Table 2). While risperidone showed comparable potency at the five pathways at which inverse agonism was observed, there were major differences in the inverse agonist potency of clozapine for the different G protein subtypes. It has the highest potency at Gα_q_ (sub-picomolar; logEC_50_: −12.31 ± 0.21) followed by Gα_i1_ (logEC_50_: −8.24 ± 0.70), Gα_11_ (logEC_50_: −8.16 ± 0.19), Gα_14_ (logEC_50_: −7.45 ± 0.30) and finally Gα_z_ (logEC_50_: −6.17 ± 0.29) (Fig. 2b and Supplementary Table 2). Of notice, the structurally-related antipsychotics clozapine and olanzapine differ significantly in their inverse agonist potency, which likely reflects the previously reported differences in the binding mode of these drugs with respect to the two serine residues in TM5 [49]. While the rank order of potency for clozapine is Gα_q_ (logEC_50_: -12.31 ± 0.21) ≫ Gα_i1_ (logEC_50_: −8.24 ± 0.70) = Gα_11_ (logEC_50_: −8.16 ± 0.19) > Gα_14_ (logEC_50_: −7.45 ± 0.30) > Gα_z_ (logEC_50_: −6.17 ± 0.29), it is Gα_11_ (logEC_50:_ −11.06 ± 0.25) ≫ Gα_q_ (logEC_50_: −9.81 ± 0.22) > Gα_14_ (logEC_50:_ −8.35 ± 0.34) ≫ Gα_i1_ (logEC_50:_ −6.87 ± 0.81) ≫ Gα_z_ (no activity) for olanzapine (Fig. 2b and Supplementary Table 2). Haloperidol, known primarily for its activity at the dopamine D_2_ receptor, has the lowest overall potency among the four antipsychotics displaying inverse agonist activity at the 5-HT_2A_R. It showed its highest potency at Gα_i1_ (logEC_50:_ −8.77 ± 0.88) and no preference (logEC_50:_ −6.70 to −7.58) among the other four G proteins engaged (Gα_q,_ Gα_11_, Gα_14_ and Gα_z_) (Fig. 2b and Supplementary Table 2).

Of interest, the relative inverse efficacy of the four compounds varied depending on the G protein subtype considered. Whereas haloperidol was the most efficacious compound for Gα_11_ and Gα_14_, the most efficacious one for Gα_q_ was risperidone. The three compounds (risperidone, clozapine and haloperidol) with inverse agonist activity at Gα_z_ were found to be equi-efficacious at this pathway (Supplementary Fig. 2). Taken together, the data unravel clear differences among clinically used antipsychotic to act as inverse agonists and displaying distinct G protein selectivity in their inverse potency and efficacy.

In addition to these compounds we also tested a clinically used antipsychotic for psychosis associated with Parkinson’s disease, pimavanserin. As shown in Supplementary Fig. 3, similar to risperidone, clozapine, olanzapine and haloperidol (Fig. 2), pimavanserin showed a robust inverse agonist efficacy on Gα_q/11_ family members. For the Gα_i/o/z_ pathways, it showed a weak inverse efficacy for Gα_i1_ but no other member of the Gα_i/o/z_ family.

### The partial agonist activity of aripiprazole and cariprazine

**Fig. 3:**
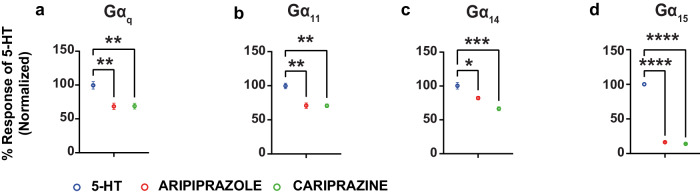
Comparison of the activities of aripiprazole and cariprazine with 5-HT in the agonist mode. Graphs comparing the maximal response of aripiprazole and cariprazine with that of 5-HT at Gα_q_ (**a**), Gα_11_ (**b**), Gα_14_ (**c**) and Gα_15_ (**d**) (mean ± SEM; *n* = 3; one-way ANOVA followed by Dunnett’s post hoc test). **p = 0.0046 for aripiprazole and **p = 0.0047 for cariprazine in **a**, **p = 0.0023 in **b**, *p = 0.0169, and ***p = 0.0007 for **c** and ****p < 0.0001 for **d**, compared to the maximal response by 5-HT at the respective pathways.

Aripiprazole and cariprazine display functional selectivity in their pattern of activation of the different G proteins. When considering the Gα_q/11_ family members, the two compounds showed partial agonist activity with relatively high efficacies at Gα_q_, Gα_11_ and Gα_14_ (their efficacies being between 67.74 ± 4.97 and 73.28 ± 3.61% of that of 5-HT) but only low efficacy partial agonists at Gα_15_ (their efficacies being only 16.29 ± 0.71 and 13.93 ± 0.53% of that of 5-HT; Fig. 2c). The two compounds were found to have similar partial agonistic activity (potency and efficacy) at each of the Gα_q_ family subtypes (Fig. 3 and Supplementary Table 3).

At Gα_i/o/z_, very weak yet statistically significant activation was detected for aripiprazole only at Gα_i1_ and Gα_z_ (Supplementary Figs. 4 and 5). Cariprazine for its part did not show statistically significant activation at the Gα_i/o/z_ family (Supplementary Figs. 4 and 5). Finally, aripiprazole showed very weak yet statistically significant recruitment of β-arrestin1 whereas cariprazine did not (Supplementary Figs. 4 and 5 and Supplementary Table 3). Together, the data show that the two compounds have a preferential partial agonistic activity at Gα_q_ *vs* Gα_i_. Differential agonistic activity was also observed toward members of the same Gα protein subtype, with Gα_q_, Gα_11_ and Gα_14_ being preferred *vs* Gα_15_ for the Gα_q/11_ family and Gα_i1_ and Gα_z_ being preferred *vs* all other Gα_i/o/z_ family members. This preference for specific G protein subtypes is reminiscent to the one observed for the 4 compounds that showed inverse agonistic activity (Fig. 2a), indicating that this selectivity is linked to the coupling preference of the receptor and not agonist-promoted pathway preferences of the ligands themselves. Yet the difference between inverse agonism and partial agonism at these G proteins between risperidone, clozapine, olanzapine and haloperidol on the one hand and aripiprazole or cariprazine on the other clearly points to a difference in the ligand-promoted regulation of subsets of G protein subtypes between these two groups of antipsychotics.

To assess whether this difference in inverse agonism and partial agonism between antipsychotics could translate in in vivo settings, we measured the levels of D-myo-inositol 1 monophosphate (IP1) in the frontal pole of the cortical lobe dissected from mice treated with risperidone, or aripiprazole or the vehicle alone for 60 min. As shown in Supplementary Fig. 6, aripiprazole promoted a statistically significant 10.5 ± 1.6% increase in IP1 levels compared to the vehicle condition, consistent with the partial agonism toward Gα_q_ that we observed in the cell-based assays. Risperidone treatment, for its part, led to a significant 9.2 ± 1.2% reduction in IP1 consistent with the inverse efficacy observed in cells. However, one cannot exclude the possibility that this decreases results from that antagonistic action of the drug inhibiting the activation resulting from endogenous 5-HT as ascertaining inverse agonism in vivo is always hampered by the possible tonic presence of endogenous agonists.

### Activity of the antipsychotics tested in antagonist mode

**Fig. 4:**
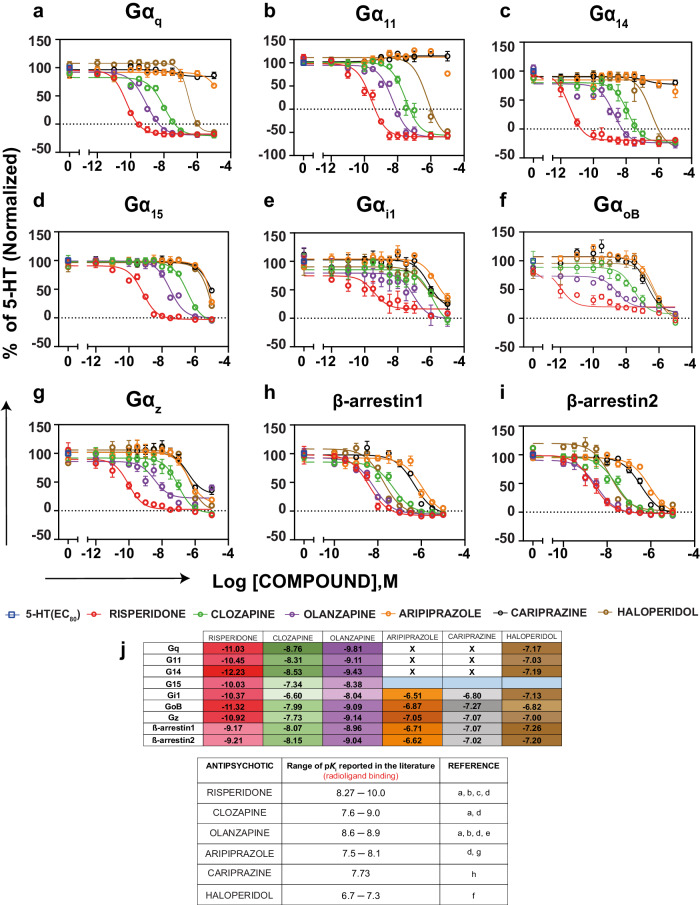
Pathway-specific antagonistic activity of the six antipsychotic drugs tested. **a-i** Concentration response curves with the activity of the six antipsychotics tested in the antagonist mode (i.e; in the presence of an EC_80_ concentration of 5-HT) using the ebBRET-based EMTA biosensors in HEK293 cells heterologously expressing the untagged 5-HT_2A_R and the biosensor (rGFP-CAAX along with p63-RlucII for Gα_q_ family, Rap1GAP-RlucII for Gα_i/o/z_, and the respective Gα subunits, as well as β-arrestin1-RlucII or β-arrestin2-RlucII and WT-GRK2 for recruitment of β-arrestins). Results are expressed as % response of 5-HT (EC_80_) at the respective pathways (normalized with respect to the response of 5-HT (EC_80_)) (mean ± SEM; *n* = 3–5). **j** Heatmap depicting the equilibrium dissociation constant (*K*_b_) of the antipsychotics calculated based on the modified Cheng-Prusoff equation as described in the methods section along with the p*K*_i_ values reported in the literature (**a** [66]; **b** [67]; **c** [68]; **d** [69]; **e** [70]; **f** [28]; **g** [56]; **h** [71]). The empty cells with a cross indicate no activity in the antagonist mode whereas cells colored blue indicate IC_50_ > 10 µM.

We tested the antipsychotics for their effect on the 5-HT (EC_80_)-mediated activation of the different G protein pathways and on the recruitment of β-arrestins. As was observed in the agonist mode, the inverse agonist activity of risperidone, clozapine, olanzapine and haloperidol could be seen at Gα_q_, Gα_11_ and Gα_14_ as reflected by the fact that the compounds not only fully antagonized the 5-HT-promoted BRET response but also promoted a BRET reduction below basal activity (Fig. 4a–c and Supplementary Table 4b). For Gα_15_, Gα_z_, Gα_i1_, Gα_oB_ and the β-arrestins, the four compounds behave as full antagonists and no inverse agonist activity could be detected (Fig. 4d-i and Supplementary Table 4b).

On account of being high efficacy partial agonists at Gα_q_, Gα_11_ and Gα_14_, aripiprazole and cariprazine did not show antagonistic activity for the 5-HT (EC_80_)-mediated activation of these pathways (Fig. 3 and Supplementary Tables 3, 4a, b). In contrast, the two compounds were low potency full antagonists for Gα_oB_, Gα_z_ and β-arrestins, and in lieu of their weak partial agonist activity at Gα_15_ and Gα_i1_, they are partial antagonists with relatively high inhibitory efficacy at these pathways (inhibiting the 5-HT promoted responses by ~80% at Gα_i1_; Figs. 3, 4 and Supplementary Table 4a, b). These data indicate that aripiprazole and cariprazine have pathway selective efficacies at the 5-HT_2A_R.

Although not a direct indication of binding affinity, the IC_50_ values obtained at the different pathways were used to calculate the apparent equilibrium dissociation constants (*K*_b_) of the antipsychotics (Fig. 4j) based on the modified Cheng-Prusoff equation [41]. Overall, the apparent affinities calculated from the functional data follow the order of measured affinities reported in the literature. However, the analyses reveal the existence of distinct functionally-derived apparent affinities for the different pathways considered, indicating that affinities derived from binding experiments cannot be used directly to predict how potent a drug will be at inhibiting a specific pathway. Good examples of this are provided by risperidone and olanzapine. Risperidone displayed a *K*_b_ 1000-times lower to inhibit Gα_q_ than to block β-arrestin recruitment. For its part, the *K*_b_ of olanzapine to inhibit Gα_q_ and inhibit the 5-HT-mediated β-arrestin recruitment were similar, but 100-fold lower than for inhibiting Gα_i1_.

**Fig. 5:**
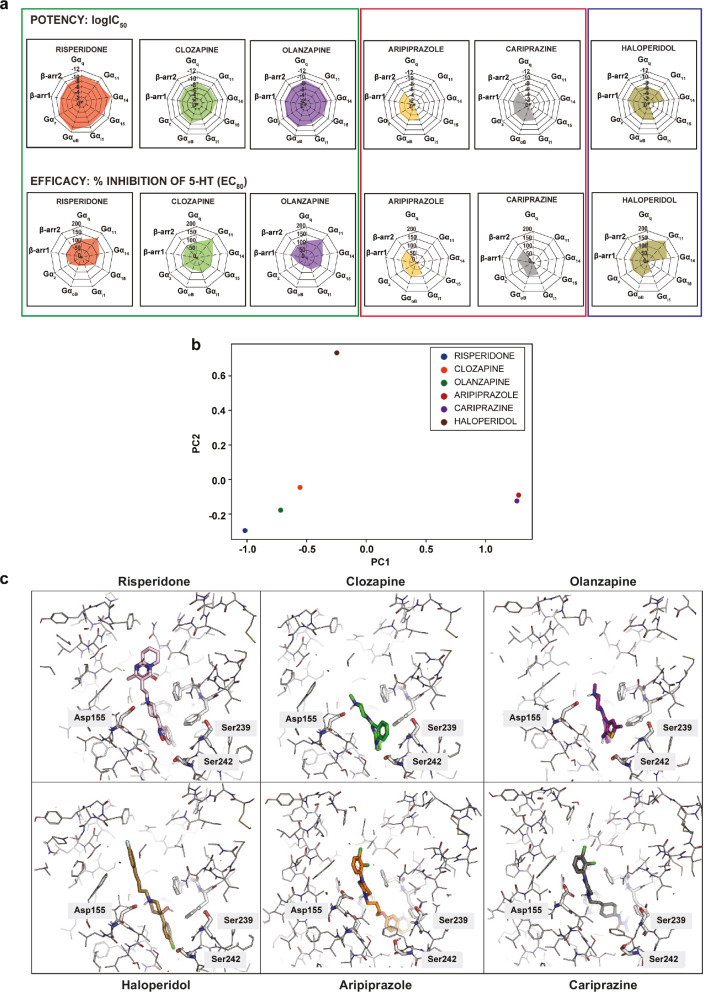
Pharmacological and binding properties of the six antipsychotic drugs tested. **a** Web representation of the potency (logIC_50_) and efficacy (% inhibition) of the six antipsychotics tested in the antagonist mode. **b** Principal component analysis of the antagonist mode (potency and efficacy) depicting the hierarchical clustering of the antipsychotics into three distinct groups. **c** Binding modes of the six antipsychotics at the 5-HT_2A_R.

Figure 5 illustrates the overall antagonist and inverse agonist transducer engagement profiles observed for the six antipsychotic drugs. The web representation allows to clearly see that the drugs can be classified into three different clusters according to their relative efficacies and potencies toward the different G protein subtypes and β-arrestins. Risperidone, clozapine and olanzapine displayed what could be considered as a balanced profile since each of the drugs showed similar potencies and efficacies among the pathways. In sharp contrast, aripiprazole and cariprazine showed marked pathway preference, having antagonistic activity only at a subset of the pathways. Haloperidol fell into a category of its own, where pathway preference could be seen only when considering its potencies for the different pathways. Of significant notice, the three clusters correspond to the classification of these antipsychotics as defined by their therapeutic profiles, namely typical (haloperidol), atypical (risperidone, clozapine and olanzapine) and third generation (aripiprazole and cariprazine). This visual clustering based on their overall signaling profiles was further confirmed using principal component analysis of the data (Fig. 5b).

Given the clear separation into three groups at the pathway level, we were wondering whether this was also borne out at the structural level. To that end, we analyzed the existing experimental structural data for risperidone (6A93) [50], aripiprazole (7VOE) [51], and cariprazine (7VOD) [51] and generated docking-derived poses for the remaining three molecules. As can be seen in Fig. 5c, the poses of olanzapine and clozapine overlay well with the X-ray structure of the risperidone-bound receptor. Their three-ring (clozapine and olanzapine) and two-ring (risperidone) systems, respectively, are positioned deep in the pocket, close to Ser239, a residue that has been described as being involved in receptor activation networks [52]. The fact that in the poses of all three ligands hydrogen bond donors or acceptors are close to the side chain of Ser239 might point to a role of this residue in their similar pathway signatures (Fig. 5a). Aripiprazole and cariprazine are positioned even deeper along the z-axis, pointing into the space between helices 5 and 6. The binding modes of these two drugs are substantially overlapping, and the residues surrounding the deepest-binding portions are again made up of residues such as Phe332 (6.44) and V333 (6.45) implicated in receptor activation [52, 53], possibly leading to a distinct conformation which could be responsible for their partial agonism. We would like to note, however, that our docking calculations also yielded alternative poses for aripiprazole and cariprazine that bound more in the direction of the extracellular space. In these poses, the dihydroquinoline and urea motifs, respectively, are involved in higher numbers of polar contacts compared to the experimental structures (Supplementary Fig. 7). Still, even in these alternative poses, aripiprazole and cariprazine are highly congruent, providing a possible rationale for their similar signaling profiles. Finally, the pose of haloperidol is located somewhat in-between the other two regions, and lacks the bulky rings close to Ser239 that is a common motif for clozapine, olanzapine and risperidone. These analyses support the notion that the binding modes also cluster in 3 different groups in line with the signaling signature.

## Discussion

The exhaustive profiling of six clinically used antipsychotics belonging to the three classes of drugs allowed us to confirm some of the already known pharmacological properties of these compounds but also revealed an unprecedented level of functional selectivity, which could have therapeutic ramifications. The most salient differences include clear preferences of the compounds in terms of their efficacy for the different pathways engaged by the receptor.

When considering the inverse efficacy of the compounds, although previous studies have reported some level of inverse efficacy mainly at the Gα_q_ pathway, our study reveals a much broader spectrum of inverse efficacies not only at Gα_q_ but also at other G proteins, but not β-arrestins. In addition, different level of inverse agonistic activity both at the efficacy and potency levels were found between the different G proteins. For example, clozapine is a 10,000-fold more potent inverse agonist at Gα_q_ compared to Gα_11_, Gα_14_, Gα_z_ and Gα_i1_, a characteristic that is not shared by the other antipsychotics with inverse efficacy. Whether this may have relevance for the distinct clinical therapeutic/side effect profile of clozapine remains to be investigated but it is worth noting that the inverse agonism of Gα_q_ by clozapine through the 5-HT_2A_R was found to potentiate Gα_i_ signaling through the 5-HT_2A_R-mGlu_2_R heterocomplex that is upregulated in schizophrenia, and to be involved in the mechanism of its antipsychotic activity [12, 54].

The only drug for which inverse efficacy at the 5-HT_2A_R has previously been observed at a G protein other than Gα_q_, namely Gα_i1_, is the 5-HT_2A_R-selective drug pimavanserin [26]. In the present study we found that four antipsychotics (risperidone, clozapine, olanzapine and haloperidol) are also inverse agonists at Gα_i1_, whereas three of them (risperidone, clozapine and haloperidol) also showed inverse efficacy for one other member of the Gα_i/o/z_ family, Gα_z_. Previous studies have implicated Gα_i1_ signaling through 5-HT_2A_R activation in the mechanism of action of hallucinogenic drugs, and a supersensitive coupling of 5-HT_2A_R to Gα_i1_ as opposed to Gα_q_ has been identified in the postmortem brain samples of schizophrenia patients. [7, 15, 26, 44].

Pimavanserin, which has been reported to be an inverse agonist at the 5-HT_2A_R/Gα_i1_ pathway [26] has been approved for Parkinson’s disease-associated hallucinations and delusions. In the present study, pimavanserin, similar to risperidone, clozapine, olanzapine and haloperidol was found to be a robust inverse agonist on Gα_q/11_ family members, whereas it showed only a weak inverse efficacy for Gα_i1_ but not for other members of the Gα_i/o/z_ family. Whether, as it was proposed for pimavanserin, the inverse agonism at Gα_i1_ of risperidone, clozapine, olanzapine and haloperidol, measured in cell-based assays, may have the potential of being therapeutically useful in ameliorating the positive symptoms of schizophrenia remains to be investigated. The fact that pimavanserin, which was originally developed as an atypical antipsychotic is generally not used as a primary drug in schizophrenia patients [55] should also be considered in that context. Interestingly, the signaling profile obtained for pimavanserin is different from those observed for the six other antipsychotics tested.

Also, of interest is the selective high partial agonist efficacy of aripiprazole and cariprazine at Gα_q_, Gα_11_ and Gα_14_ compared to their low efficacy partial agonist activity at Gα_15_ and the mainly neutral antagonist activity at the Gα_i/o/z_ family members and β-arrestins. Although previous studies have recognized these two D_2_/D_3_ and 5-HT_1A_ receptor-preferring antipsychotics for their antagonist activity at the 5-HT_2A_R [56, 57], other studies have also detected partial agonist activity [51], thus underlying the fact that the cell type and pharmacological assay play a critical role, especially with regard to distinguishing partial agonism from neutral antagonism. The significant differences that we observe in the efficacy among the distinct G protein signaling pathways might be related to the inherent coupling efficiency of the 5-HT_2A_R for the different G protein subtypes and the conformational state sampled by the ligand-bound receptor when coupled to different G proteins. The deviation of the activity at Gα_15_ from the other members of the Gα_q_ family most likely reflects the distinct characteristics of Gα_15_ compared to the other members of the Gα_q/11_ family [20, 58].

Although 5-HT_2A_R agonists are associated with hallucinogenic properties and that aripiprazole and cariprazine are high efficacy partial agonists on some pathways (Gα_q_, Gα_11_ and Gα_14_), the general absence of agonist efficacy at the Gα_i/o/z_ family members could be one of the reasons why these compounds possess minimum risk of hallucinatory side effects. However, it would be pertinent to note here that the experimental system of the present study does not have heterologously expressed mGlu_2_R, the heteromeric partner of the 5-HT_2A_R shown to be contributing to the Gα_i_ signaling through 5-HT_2A_R in neurons [11].

In line with previous studies showing pharmacological and behavioral differences, especially between the typical antipsychotic haloperidol and the atypical drugs risperidone, clozapine and olanzapine [3, 59–65], our study unravels critical differences in the signaling profiles between antipsychotics belonging to the three different classes of clinically used antipsychotics. Remarkably, the pathway-specific equilibrium dissociation constants (log*K*_B_) highlight the differences in the capacity of the antipsychotics to engage the different signaling pathways. This illustrates the power of profiling the pathway preference and functional selectivity of compounds to classify drugs in different categories that may have clinical relevance and provide predictive value in drug discovery programs.

The distinct binding modes and docking poses of the six antipsychotic drugs along with the variations in the interactions with key amino acids in and around the binding pocket of the receptor is consistent with the fact that these compounds have different functional selectivity profiles. The similarities in the docking poses observed between antipsychotics belonging to a specific clinical category (atypical: risperidone, clozapine and olanzapine), (third generation: aripiprazole and cariprazine) that differed from haloperidol (typical) provides further insights in structural determinants of their distinct profiles both in clinical setting and signaling activities.

A limitation of the present study is that the signaling profile of the different antipsychotics at the 5-HT_2A_R was carried out in HEK293 heterologously expressing the receptor and not in the neuronal cells which are the targets for these drugs. In addition to the cell type specificities, at 1675 fmol/mg protein, it is difficult to ascertain that the receptor expression level is within the physiological range as there is no reliable estimate of the receptor density at the synapse of the physiologically relevant nuclei. In any case, the results provide a useful profiling of the possible signaling repertoire of the 5-HT_2A_R and of the distinct abilities of different antipsychotics to selectively regulate the signaling pathways of this repertoire. Validating the signaling profiles of the different antipsychotics in native tissues and test their impact on the therapeutic outcomes will await further in vivo experiments that will selectively be designed for this purpose. Yet, our observation that the partial agonisms of aripiprazole and inverse agonism of risperidone on the Gα_q_ pathway could be confirmed by an elevation and decrease of IP1 levels, respectively, in the frontal cortical lobe of mice treated with these two drugs supports the possibility to translate our findings into physiological context.

A formal extrapolation of the functional selectivity profile of the antipsychotic drugs to their effect on specific symptoms of schizophrenia or side effects is beyond the scope of the present study. Yet the large data set generated in this simplified system have the distinct advantage of propounding functional selectivity at the level of individual G protein and β-arrestin pathways, and represent a stepping stone towards dissecting the signaling pathways associated with the therapeutic effects of antipsychotics from those leading to side effects.

## Supplementary information

Supplementary Information

## Data Availability

All data needed to evaluate the conclusions in the paper are present in the paper and/or the Supplementary Materials.

## Materials availability

Some of the biosensors used in present study are protected by patents, but all are available for academic research under regular material transfer agreement (MTA) upon request to MB.
