# Supplementary material for: Pharmacological fingerprint of antipsychotic drugs at the serotonin 5-HT2A receptor
Source: Mol Psychiatry. 2024 Apr 2;29(9):2753–64. doi: 10.1038/s41380-024-02531-7 (PMC11420065; doi:10.1038/s41380-024-02531-7)
Supplement: Supplementary file 1 — Supplementary Information [file 41380_2024_2531_MOESM1_ESM.docx]

**Pharmacological fingerprint of antipsychotic drugs at the serotonin 5-HT_2A_ receptor**

Supriya A. Gaitonde, Ph.D.^1^, Charlotte Avet, Ph.D.^1^, Mario de la Fuente Revenga, Ph.D.^2^, Elodie Blondel-Tepaz, Ph.D.^1^, Aida Shahraki, Ph.D.^3^, Adrian Morales Pastor, M.S.^4^, Valerij Talagayev, M.Sc.^3^, Patricia Robledo, Ph.D.^5^, Peter Kolb, Ph.D.^3^, Jana Selent, Ph.D.^4^, Javier González-Maeso, Ph.D.^2^, Michel Bouvier, Ph.D.^1,6^

^1^Institute for Research in Immunology and Cancer (IRIC), Department of Biochemistry and Molecular Medicine, Université de Montréal, Montréal, Québec H3T 1J4, Canada.

^2^Department of Physiology and Biophysics, School of Medicine, Virginia Commonwealth University, Richmond, VA, 23298, USA.

^3^Department of Pharmaceutical Chemistry, Philipps-Universität Marburg, Marbacher Weg 8, 35032 Marburg, Germany.

^4^Research Programme on Biomedical Informatics (GRIB), IMIM-Hospital del Mar Medical Research Institute, Barcelona, 08003 Spain.

^5^Integrative Pharmacology and Systems Neuroscience Research Group, IMIM-Hospital del Mar Medical Research Institute, Barcelona, 08003 Spain.

^6^To whom correspondence may be addressed:

Michel Bouvier

IRIC | Université de Montréal

C.P. 6128, succursale Centre-ville

Montréal (Québec) H3C 3J7

CANADA

Tel: 514-343-6319

Fax: 513-343-7780

[michel.bouvier@umontreal.ca](mailto:michel.bouvier@umontreal.ca) (M.B.).

**Running title: Fingerprint of antipsychotics at 5-HT_2A_**

**SUPPLEMENTARY INFORMATION (SI)**

**SUPPLEMENTARY FIGURES:**

**Supplementary Fig. 1:** The signaling profile of 5-HT at the 5-HT_2A_ receptor in HEK293 cells.

**Supplementary Fig. 2:** Inverse agonist activity of risperidone, clozapine, olanzapine and haloperidol.

**Supplementary Fig. 3:** Concentration response curves showing the activation profile of pimavanserin in the agonist mode and antagonist mode.

**Supplementary Fig. 4:** Concentration response curves showing the activation of the Gα_i/o/z_ family and βarrestin1/2 recruitment by 5-HT, aripiprazole and cariprazine.

**Supplementary Fig. 5:** Graphs comparing the baseline and maximal response for Gα_i/o/z_ family activation and βarrestin1/2 recruitment mediated by 5-HT_2A_R in response to aripiprazole and cariprazine.

**Supplementary Fig. 6:** Quantification of the change in the IP1 production in the frontal pole of the cortical lobe of mice treated with risperidone or aripiprazole.

**Supplementary Fig. 7:** Alternative poses for aripiprazole and cariprazine obtained from docking studies.

**Supplementary Table 1:** logEC_50_ and logEC_80_ of 5-HT at the different G protein pathways and for recruitment of the β-arrestins.

**Supplementary Table 2:** Inverse agonist activity of antipsychotics at the G protein pathways.

**Supplementary Table 3:** Partial agonist activity of aripiprazole and cariprazine at the G protein pathways (normalized as % response of 5-HT).

**Supplementary Table 4a:** The potency (logIC_50_) of the six antipsychotics for the inhibition of the 5-HT (EC_80_)-mediated activation of the different G protein pathways and recruitment of β-arrestin1 and β-arrestin2.

**Supplementary Table 4b:** The efficacy (% inhibition) of the six antipsychotics for the inhibition of the 5-HT (EC_80_)-mediated activation of the different G protein pathways and recruitment of β-arrestin1 and β-arrestin2.

**Supplementary Table 5:** The equilibrium dissociation constant (log*K*_B_) of the antipsychotics for the different pathways calculated based on the modified Cheng-Prusoff equation.

**SUPPLEMENTARY INFORMATION (SI)**

**SUPPLEMENTARY FIGURES:**


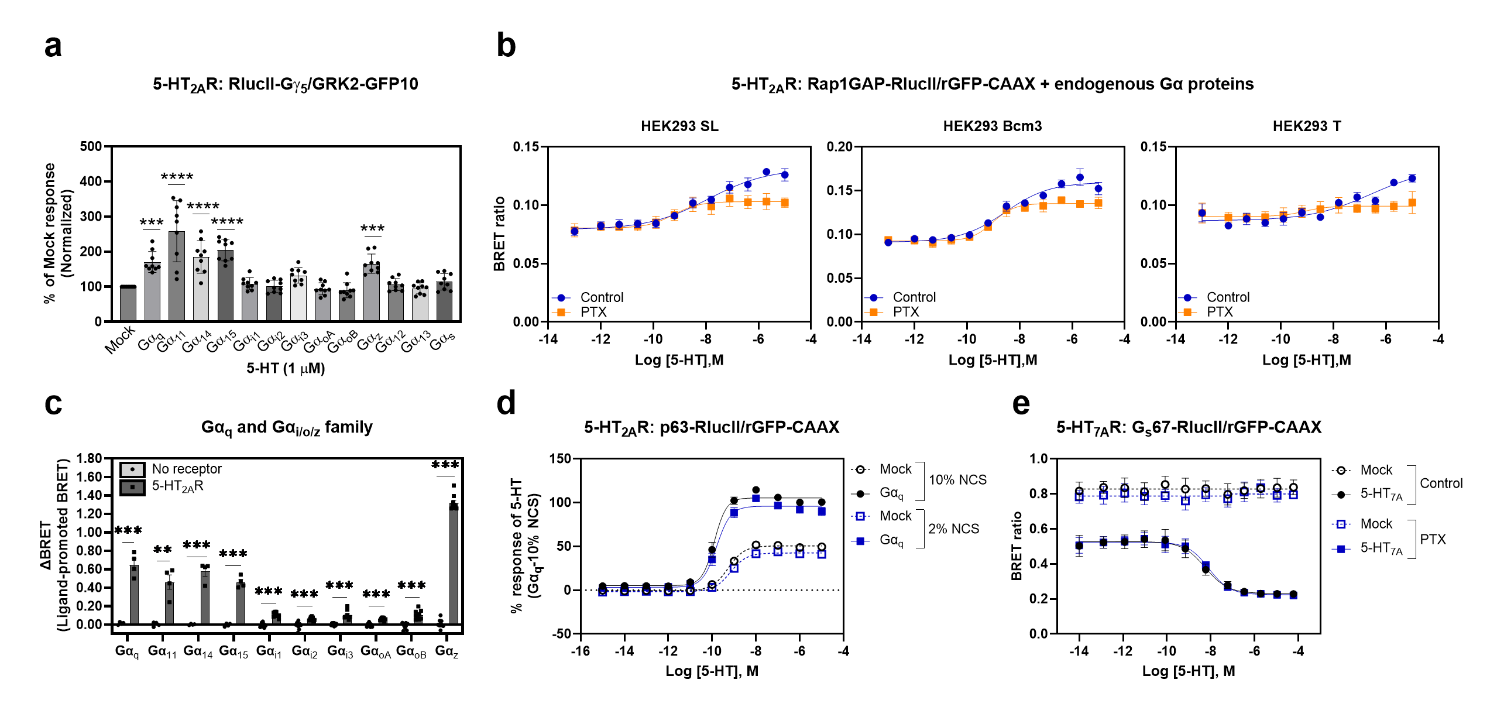


**Supplementary Fig. 1** The signaling profile of 5-HT at the 5-HT_2A_ receptor in HEK293 cells. **a** The complete G protein activation profile of 5-HT (1 µM; 15 min) with HEK293 cells heterologously expressing the untagged 5-HT_2A_ receptor and the biosensor (RlucII-G_γ5_/GRK2-GFP10, Gβ_1_ and the respective Gα subunits). Results are expressed as BRET ratio in % of mock condition (in the absence of heterologously expressed Gα subunit) (mean ± SEM; *n* = 3; one-way ANOVA followed by Dunnett’s post hoc: ****p* < 0.001, and *****p* < 0.0001 compared to the mock condition). **b** Concentration response curves showing activation of the endogenous Gα_i/o/z_ family with the EMTA biosensor in three different background of HEK293 cells (SL, Bcm3 or T) heterologously expressing the untagged 5-HT_2A_ receptor and the biosensor (Rap1Gap-RlucII/rGFP-CAAX) in the absence and presence of pertussis toxin (PTX). The inhibition of the response indicates direct response through the Gα_i/o_ family and the remaining PTX insensitive signal, corresponding to endogenous Gα_z_ activation, was found to be from 48 ± 5, 61 ± 7 and 40 ± 8 % of the global Gα_i/o/z_ responses observed in HEK293 SL, Bcm3 and T cells, respectively. Results are expressed as BRET ratio (mean ± SEM; *n* = 3). **c** Bar graphs showing activation of the Gα_q_ and Gα_i/o/z_ families in response to 5-HT (1µM; 15 min) with the EMTA biosensors (p63-RlucII/rGFP-CAAX and Rap1Gap-RlucII/rGFP-CAAX, respectively), in HEK293 cells heterologously expressing or not the 5-HT_2A_R. Results are expressed as ΔBRET (ligand-promoted BRET; mean ± SEM; *n* = 4-9; Unpaired t-test (two-tailed) : ***p* < 0.01 and ****p* < 0.001 compared to respective no receptor condition). **d** Concentration response curves showing activation of the endogenous (mock) or overexpressed Gα_q_ proteins in HEK293 cells heterologously expressing the 5-HT_2A_ receptor and the EMTA biosensor (p63-RlucII/rGFP-CAAX) and cultivated in 10% NCS or starved in 2% NCS the night before BRET experiment. Results are expressed as BRET ratio in % of maximal response obtained for Gα_q_-10% NCS cells (mean ± SEM; *n* = 3-4). **e** Concentration response curves illustrating 5-HT-promoted Gα_s_ activation in HEK293 cells transfected (closed symbols) or not (open symbols) with a 5-HT_7A_ encoding plasmid in the presence of the EMTA biosensor (G_s_67-RlucII/rGFP-CAAX. No activation could be detected in the absence of heterologously expressed 5-HT_7A_. To exclude that the lack of response could result from the activation of Gα_i/o_ by another 5-HT receptor, cells were treated or not with PTX. Again, a Gα_s_ response could be observed only in cells heterologously expressing 5-HT_7A_ Results are expressed as BRET ratio (mean ± SEM; *n* = 3).

**
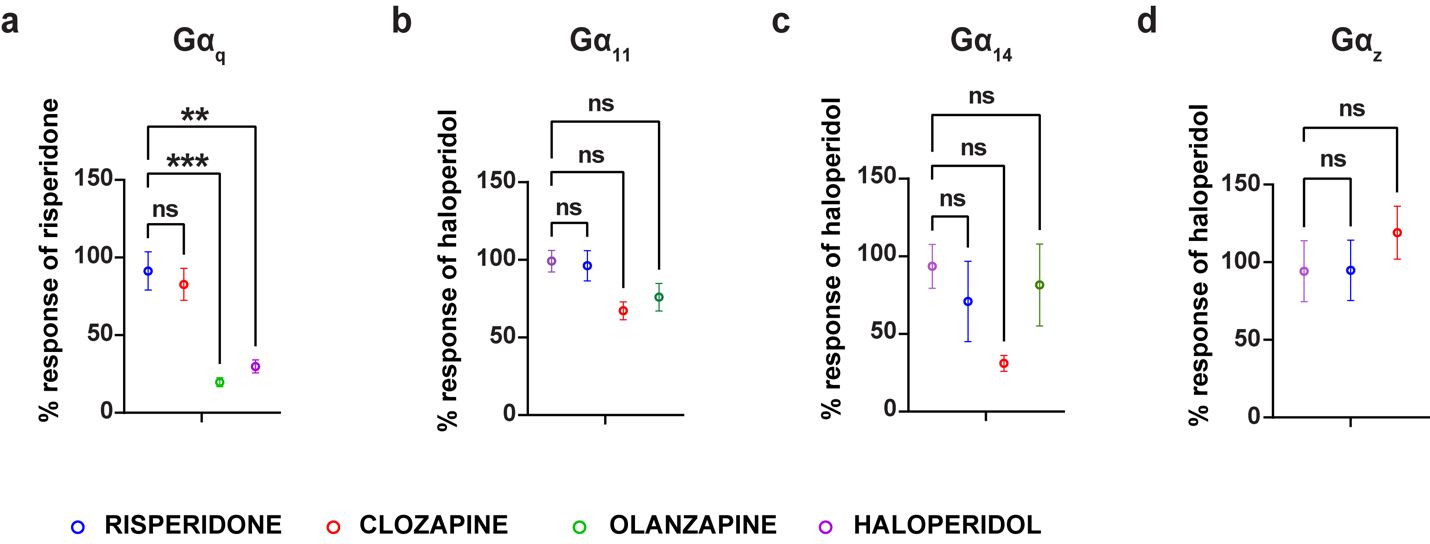
**

**Supplementary Fig. 2** Inverse agonist activity of risperidone, clozapine, olanzapine and haloperidol. **a** Graph represents the efficacy of the inverse agonist activity at Gα_q_ (**a**), Gα_11_ (**b**), Gα_14_ (**c**) and Gα_z_ (**d**), normalized with respect to the efficacy of risperidone (**a**, mean ± SEM; *n* = 3) or haloperidol (**b-d,** mean ± SEM; *n* = 3-5). Statistical analysis: one-way ANOVA followed by Dunnett’s post hoc test compared to risperidone for Gα_q_ and haloperidol for Gα_11_, Gα_14_ and Gα_z_ (***p* = 0.0022, ****p* = 0.0008, ns: not significant).


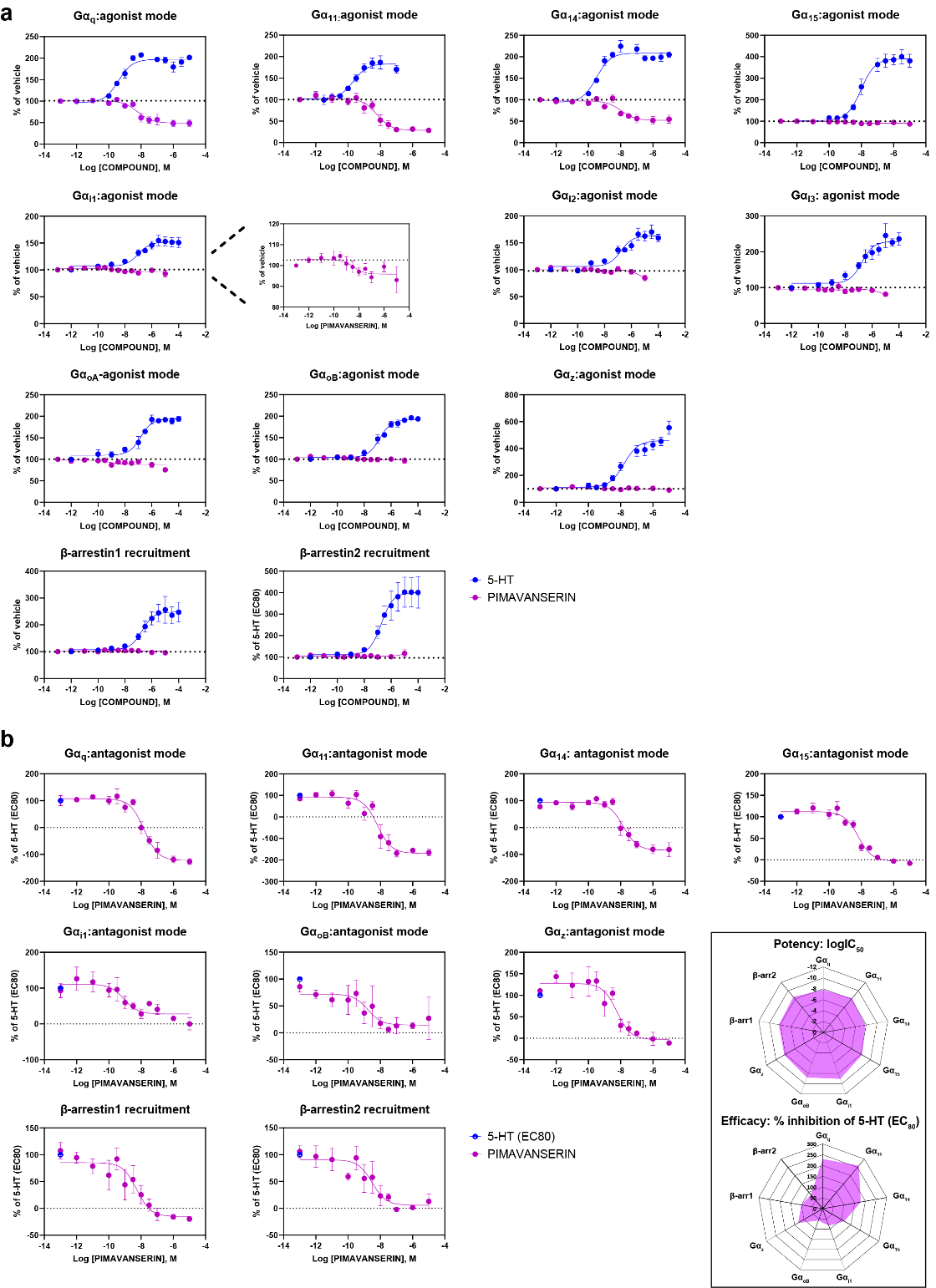


**Supplementary Fig. 3** Concentration response curves showing the activation profile of pimavanserin in the agonist mode (**a**) and antagonist mode (**b**) using the EMTA biosensors in HEK293 cells heterologously expressing the untagged 5-HT_2A_ receptor and the biosensors (p63-RlucII/rGFP-CAAX for Gα_q_ family or Rap1GAP-RlucII/rGFP-CAAX for Gα_i/o/z_ family with the respective Gα subunits, and β-arrestin1-RlucII or β-arrestin2-RlucII/rGFP-CAAX). Results are the BRET ratio in % of vehicle for agonist mode and in % an EC_80_ of 5-HT for antagonist mode (mean ± SEM; *n* = 3-5). Note: The inset in (**a**) indicated by the dotted line representing the Gα_i1_ inverse agonism is the same curve that the one shown in the “Gα_i1_: agonist mode” on the left graph, but with a modified scale for Y axis. *Bottom right inset* in (**b**): Web representation of the potency (logIC_50_) and efficacy (% inhibition) of the pimavanserin tested in the antagonist mode.

**
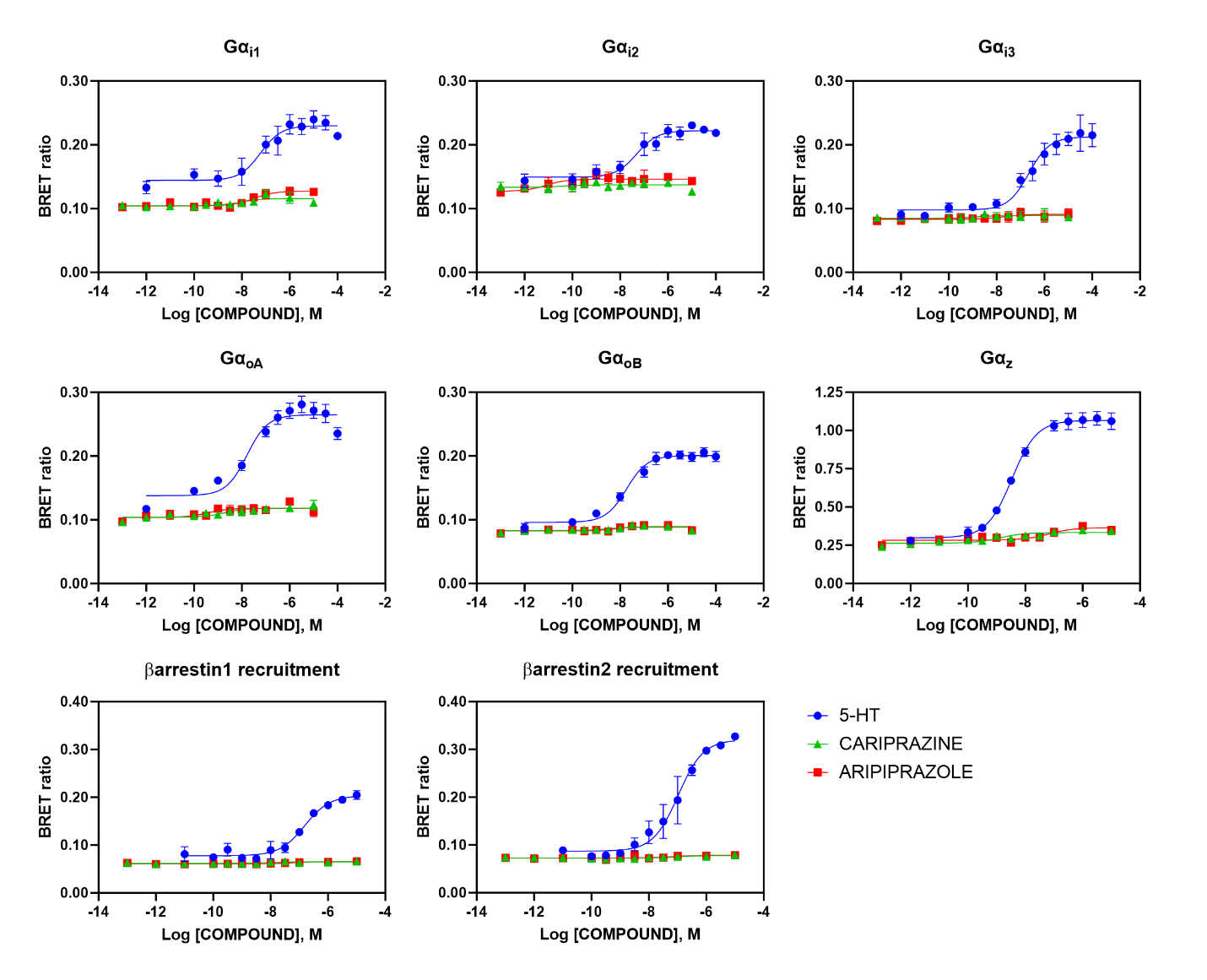
**

**Supplementary Fig. 4** Concentration response curves showing the activation of the Gα_i/o/z_ family and βarrestin1/2 recruitment by 5-HT, aripiprazole and cariprazine using the EMTA biosensors in HEK293 cells heterologously expressing the 5-HT_2A_ receptor and the biosensor (Rap1GAP-RlucII/rGFP-CAAX with the respective Gα subunits or βarrestin1/2-RlucII/rGFP-CAAX). Results are expressed as BRET ratio (mean ± SEM; *n* = 3-4).

**
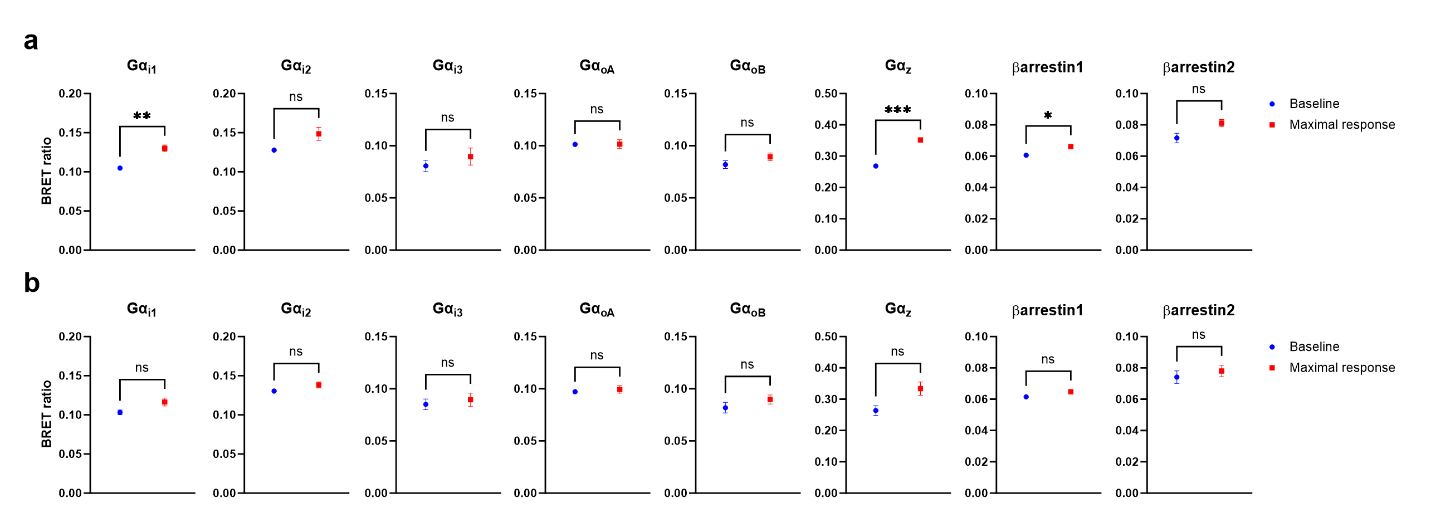
**

**Supplementary Fig. 5** Graphs comparing the baseline and maximal response for Gα_i/o/z_ family activation and βarrestin1/2 recruitment mediated by 5-HT_2A_R in response to aripiprazole (**a**) and cariprazine (**b**). Data are expressed as BRET ratio (mean ± SEM; n = 3-4; Unpaired t-test (two-tailed): *p < 0.1, **p < 0.01 and ***p < 0.001 compared to respective baseline condition).


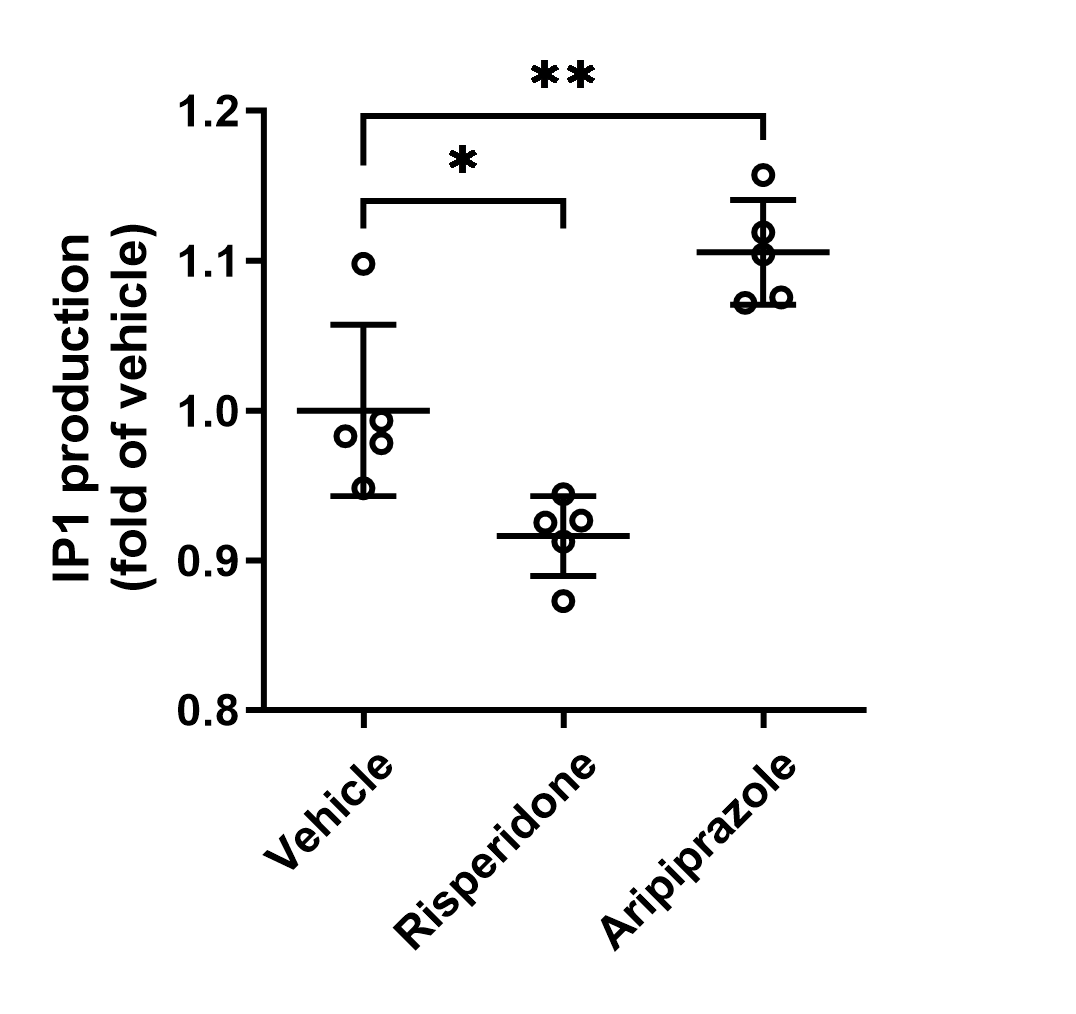


**Supplementary Fig. 6** Quantification of the change in the IP1 production in the frontal pole of the cortical lobe of mice treated with risperidone (3 mg/Kg, 1h) or aripiprazole (2 mg/Kg, 1h). Data are normalized in fold of IP1 production in vehicle-treated mice (*n* = 5 animals per treatment condition; one-way ANOVA followed by Dunnett’s post hoc: compared to vehicle at 1h: **p* < 0.1 and ***p* < 0.01 compared to vehicle).


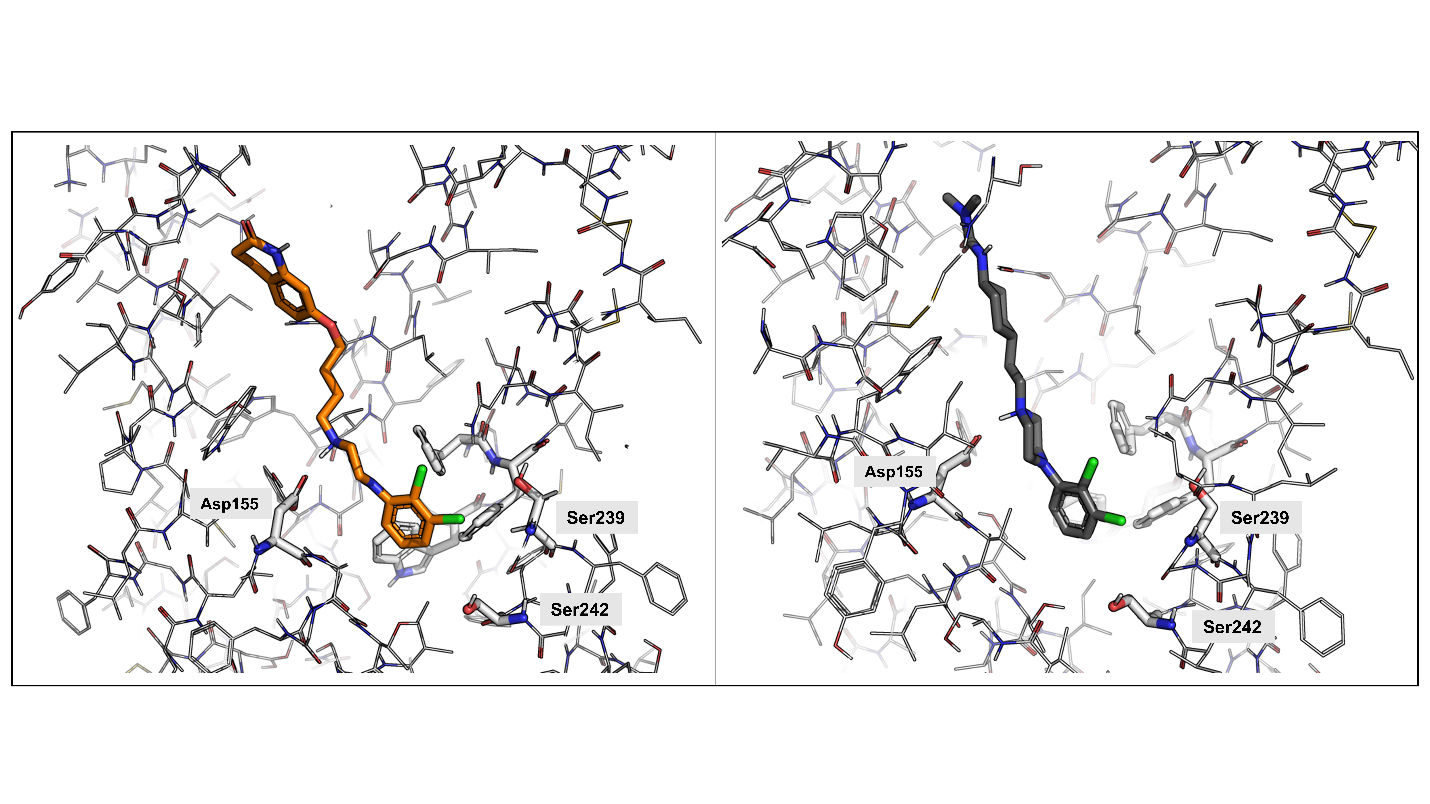


**ARIPIPRAZOLE**

**CARIPRAZINE**

**Supplementary Fig. 7** Alternative poses for aripiprazole (left) and cariprazine (right), obtained from docking studies.

**SUPPLEMENTARY TABLES:**

**Supplementary Table 1.** logEC_50_ and logEC_80_ of 5-HT at the different G protein pathways and for recruitment of the β-arrestins

| Pathway | logEC_50_ ± SEM | logEC_80_ ± SEM |
| --- | --- | --- |
| Gα_q_ | -9.49 ± 0.14 | -8.89 ± 0.14 |
| Gα_11_ | -9.81 ± 0.09 | -9.20 ± 0.09 |
| Gα_14_ | -9.47 ± 0.07 | -8.86 ± 0.07 |
| Gα_15_ | -8.35 ± 0.05 | -7.75 ± 0.05 |
| Gα_i1_ | -7.26 ± 0.21 | -6.66 ± 0.21 |
| Gα_i2_ | -7.66 ± 0.22 | -7.06 ± 0.22 |
| Gα_i3_ | -8.02 ± 0.24 | -7.41 ± 0.24 |
| Gα_oA_ | -7.79 ± 0.19 | -7.19 ± 0.19 |
| Gα_oB_ | -7.65 ± 0.10 | -7.05 ± 0.10 |
| Gα_z_ | -8.472 ± 0.05 | -7.87 ± 0.05 |
| β-arrestin1 | -6.812 ± 0.09 | -6.21 ± 0.09 |
| β-arrestin2 | -7.020 ± 0.15 | -6.42 ± 0.15 |

**Supplementary Table 2.** Inverse agonist activity of antipsychotics at the G protein pathways

|  | Risperidone | Clozapine | Olanzapine | Haloperidol |
| --- | --- | --- | --- | --- |
| Gα_q_ | -12.56 ± 0.14 | -12.31 ± 0.21 | -9.81 ± 0.22 | -7.58 ± 0.21 |
| Gα_11_ | -11.35 ± 0.18 | -8.16 ± 0.19 | -11.06 ± 0.25 | -6.77 ± 0.21 |
| Gα_14_ | -12.20 ± 0.28 | -7.45 ± 0.30 | -8.35 ± 0.34 | -7.13 ± 0.84 |
| Gα_z_  Gα_i1_ | -9.86 ± 0.36  -10.61 ± 0.42 | -6.17 ± 0.29  -8.24 ± 0.70 | NA  -6.87 ± 0.81 | -6.70 ± 0.49  -8.77 ± 0.29 |

logEC_50_ ± SEM

NA: no activity

**Supplementary Table 3.** Partial agonist activity of aripiprazole and cariprazine at the G protein pathways (normalized as % response of 5-HT)

|  | Aripiprazole | | Cariprazine | |
| --- | --- | --- | --- | --- |
|  | logEC_50_ | E_max_ | logEC_50_ | E_max_ |
| Gα_q_ | -7.87 ± 0.15 | 67.74 ± 4.97 | -7.70 ± 0.13 | 67.99 ± 4.29 |
| Gα_11_ | -8.56 ± 0.13 | 70.75 ± 4.97 | -8.42 ± 0.08 | 70.72 ± 4.97 |
| Gα_14_ | -6.94 ± 0.10 | 73.28 ± 3.61 | -7.72 ± 0.09 | 68.10 ± 3.05 |
| Gα_15_ | -7.59 ± 0.09 | 16.29 ± 0.71 | -7.61 ± 0.08 | 13.93 ± 0.53 |
| Gα_i1_ | -7.89 ± 0.37 | 26.30 ± 3.70 | -8.91 ± 0.59 | 12.72 ± 3.42 |
| Gα_i2_ | -7.73 ± 0.27 | 17.15 ± 1.93 | -8.92 ± 0.22 | 11.72 ± 2.50 |
| Gα_i3_ | -7.47 ± 0.26 | 21.85 ± 2.90 | -7.76 ± 0.32 | 16.05 ± 2.57 |
| Gα_oA_ | -9.37 ± 0.37 | 11.59 ± 2.90 | -8.56 ± 0.46 | 11.59 ± 2.44 |
| Gα_oB_ | -8.43 ± 0.38 | 6.64 ± 1.46 | -8.17 ± 0.74 | 4.32 ± 2.16 |
| Gα_z_ | -7.18 ± 0.30 | 10.64 ± 1.68 | -8.90 ± 0.21 | 9.02 ± 0.88 |
| βarrestin1 | NA | NA | NA | NA |
| βarrestin2 | NA | NA | NA | NA |

logEC_50_ ± SEM

E_max_ (%) ± SEM

NA: no activity

**Supplementary Table 4a.** The potency (logIC_50_) of the six antipsychotics for the inhibition of the 5-HT (EC_80_)-mediated activation of the different G protein pathways and recruitment of β-arrestin1 and β-arrestin2

|  | Risperidone | Clozapine | Olanzapine | Aripiprazole | Cariprazine | Haloperidol |
| --- | --- | --- | --- | --- | --- | --- |
| Gα_q_ | -10.27 ± 0.05 | -8.01 ± 0.07 | -9.06 ± 0.06 | NA | NA | -6.42 ± 0.08 |
| Gα_11_ | -9.58 ± 0.08 | -7.53 ± 0.08 | -8.29 ± 0.08 | NA | NA | -6.22 ± 0.11 |
| Gα_14_ | -11.46 ± 0.13 | -7.90 ± 0.12 | -8.77 ± 0.12 | NA | NA | -6.48 ± 0.15 |
| Gα_15_ | -9.17 ± 0.07 | -6.47 ± 0.10 | -7.52 ± 0.09 | logIC50 >10µM | logIC50 >10µM | logIC50 >10µM |
| Gα_i1_ | -9.44 ± 0.28 | -5.94 ± 0.28 | -7.05 ± 0.29 | -5.71 ± 0.28 | -6.13 ± 0.26 | -6.45 ± 0.33 |
| Gα_oB_ | -12.01 ± 0.29 | -7.52 ± 0.16 | -8.56 ± 0.15 | -6.35 ± 0.16 | -6.74 ± 0.16 | -6.31 ± 0.20 |
| Gα_z_ | -10.03 ± 0.13 | -7.02 ± 0.15 | -8.46 ± 0.21 | -6.40 ± 0.19 | -6.42 ± 0.24 | -6.34 ± 0.19 |
| βarrestin1 | -8.42 ± 0.09 | -7.29 ± 0.09 | -8.22 ± 0.07 | -5.97 ± 0.16 | -6.32 ± 0.10 | -7.74 ± 0.07 |
| βarrestin2 | -8.64 ± 0.10 | -7.56 ± 0.11 | -8.47 ± 0.08 | -6.06 ± 0.11 | -6.45 ± 0.07 | -7.83 ± 0.09 |

logIC_50_ ± SEM

NA: no activity

**Supplementary Table 4b.** The efficacy (% inhibition) of the six antipsychotics for the inhibition of the 5-HT (EC_80_)-mediated activation of the different G protein pathways and recruitment of β-arrestin1 and β-arrestin2

|  | Risperidone | Clozapine | Olanzapine | Aripiprazole | Cariprazine | Haloperidol |
| --- | --- | --- | --- | --- | --- | --- |
| Gα_q_ | 113.80 ± 2.53 | 101.70 ± 3.21 | 109.60 ± 3.02 | NA | NA | 134.50 ± 5.52 |
| Gα_11_ | 157.70 ± 6.25 | 157.40 ± 6.84 | 154.60 ± 5.50 | NA | NA | 175.90 ± 10.71 |
| Gα_14_ | 115.80 ± 6.89 | 102.70 ± 6.17 | 102.00 ± 5.62 | NA | NA | 124.10 ± 9.20 |
| Gα_15_ | 93.38 ± 3.20 | 105.00 ± 5.24 | 98.75 ± 4.51 | >70% | > 50% | >80% |
| Gα_i1_ | 58.21 ± 7.67 | 97.59 ± 16.42 | 79.78 ± 11.97 | 81.08 ± 16.91 | 84.84 ± 12.48 | 62.88 ± 10.18 |
| Gα_oB_ | 66.55 ± 11.70 | 81.07 ± 6.73 | 54.86 ± 3.82 | 105.70 ± 8.27 | 97.86 ± 7.66 | 98.32 ± 10.19 |
| Gα_z_ | 87.39 ± 4.97 | 96.01 ± 7.35 | 63.75 ± 6.12 | 88.97 ± 8.43 | 73.31 ± 8.67 | 107.00 ± 10.43 |
| βarrestin1 | 105.90 ± 4.36 | 89.36 ± 3.53 | 97.99 ± 3.28 | 101.00 ± 10.03 | 104.60 ± 6.03 | 114.3 ± 3.67 |
| βarrestin2 | 101.50 ± 4.79 | 98.90 ± 4.82 | 93.39 ± 3.34 | 100.30 ± 6.43 | 100.20 ± 3.78 | 117.40 ± 4.69 |

% inhibition ± SEM

NA: no activity

**Supplementary Table 5.** The equilibrium dissociation constant (log*K*_B_) of the antipsychotics for the different pathways calculated based on the modified Cheng-Prusoff equation

|  | Risperidone | Clozapine | Olanzapine | Aripiprazole | Cariprazine | Haloperidol |
| --- | --- | --- | --- | --- | --- | --- |
| Gα_q_ | -11.03 ± 0.05 | -8.06 ± 0.03 | -9.81 ± 0.05 | NA | NA | -7.17 ± 0.03 |
| Gα_11_ | -10.45 ± 0.15 | -8.31 ± 0.09 | -9.11 ± 0.03 | NA | NA | -7.03 ± 0.07 |
| Gα_14_ | -12.23 ± 0.08 | -8.53 ± 0.15 | -9.43 ± 0.19 | NA | NA | -7.19 ± 0.05 |
| Gα_15_ | -10.03 ± 0.01 | -7.34 ± 0.07 | -8.38 ± 0.06 | ND | ND | -4.72 ± 0.84 |
| Gα_i1_ | -10.37 ± 1.25 | -6.60 ± 0.10 | -8.04 ± 0.57 | -6.51 ± 0.39 | -6.80 ± 0.16 | -7.13 ± 0.11 |
| Gα_oB_ | -11.32 ± 1.09 | -7.99 ± 0.24 | -9.09 ± 0.17 | -6.87 ± 0.08 | -7.27 ± 0.11 | -6.82 ± 0.08 |
| Gα_z_ | -10.92 ± 0.27 | -7.73 ± 0.17 | -9.14 ± 0.38 | -7.05 ± 0.04 | -7.07 ± 0.05 | -7.00 ± 0.05 |
| βarrestin1 | -9.17 ± 0.08 | -8.07 ± 0.13 | -8.96 ± 0.06 | -6.71 ± 0.05 | -7.07 ± 0.04 | -7.26 ± 0.02 |
| βarrestin2 | -9.21 ± 0.21 | -8.15 ± 0.10 | -9.04 ± 0.04 | -6.62 ± 0.04 | -7.02 ± 0.07 | -7.20 ± 0.08 |

log*K*_B_ ± SEM

NA: no activity

ND: not determinable
